# Long-term continuous *N*-carbamylglutamate treatment in frequently decompensated propionic acidemia: a case report

**DOI:** 10.1186/s13256-018-1631-1

**Published:** 2018-04-22

**Authors:** Albina Tummolo, Livio Melpignano, Antonella Carella, Anna Maria Di Mauro, Elvira Piccinno, Marcella Vendemiale, Federica Ortolani, Stefania Fedele, Maristella Masciopinto, Francesco Papadia

**Affiliations:** Metabolic Diseases and Clinical Genetics Unit, Children’s Hospital Giovanni XXIII, Via Amendola 207, 70126 Bari, Italy

**Keywords:** Propionic acidemia, Hyperammonemia, *N*-carbamylglutamate, Long-term treatment

## Abstract

**Background:**

Propionic acidemia is a rare autosomal recessive inherited metabolic disorder that can inhibit the synthesis of *N*-acetylglutamate, the obligatory activator in urea synthesis, leading to hyperammonemia. *N*-carbamylglutamate ameliorates hyperammonemia in decompensated propionic acidemia. The effects of long-term continuous *N*-acetylglutamate administration in such patients are unknown. We report our clinical experience with continuous administration of *N*-acetylglutamate for 6 years in a patient with propionic acidemia frequently presenting with hyperammonemia.

**Case presentation:**

A male Caucasian patient with frequently decompensated propionic acidemia and hyperammonemia was admitted 78 times for acute attacks during the first 9 years of his life. Continuous daily treatment with oral *N*-carbamylglutamate 100 mg/kg (50 mg/kg after 6 months) was initiated. During 6 years of treatment, he had a significant decrease in his mean plasma ammonia levels (75.7 μmol/L vs. 140.3 μmol/L before *N*-carbamylglutamate therapy, *p* < 0.005 [normal range 50–80 μmol/L]) and fewer acute episodes (two in 6 years).

**Conclusion:**

Our results suggest a benefit of *N*-acetylglutamate administration outside the emergency setting. If this observation is confirmed, future studies should aim to optimize the dosage and explore effects of the dosage requirements on other drugs and on protein tolerance.

## Background

Propionic acidemia (PA) is a rare autosomal recessive inherited metabolic disorder caused by a deficiency of propionyl-coenzyme A (CoA) carboxylase, which catalyzes the conversion of propionyl-CoA to methylmalonyl-CoA [1]. This defect results in the accumulation of potentially toxic metabolites that can inhibit enzymes in the tricarboxylic acid and urea cycles, causing ketoacidosis and hyperammonemia, respectively [1]. Hyperammonemia occurs when accumulated propionyl compounds inhibit synthesis of *N*-acetylglutamate (NAG), the obligatory allosteric activator of carbamoyl phosphate synthetase 1, the entry step in the urea cycle [2, 3]. Hyperammonemia and metabolic acidosis are major clinical issues during decompensation in patients with PA. Neonatal onset and multiple episodes of metabolic decompensation are associated with severe neurological outcomes [4]. *N*-carbamylglutamate (NCG), a synthetic analogue of NAG, augments ureagenesis and decreases plasma ammonia in patients with decompensated PA when added to classical treatment for managing acute metabolic decompensation [5]. However, the effect of continuous long-term NCG treatment on the rate and severity of hyperammonemia secondary to PA is not known. We present a patient with PA characterized by frequent severe acute decompensations who was treated continuously with NCG for 6 years.

## Case presentation

A male Caucasian patient, now 15 years of age, first came to medical attention at 2 days of age because of vomiting, lethargy, and metabolic acidosis. Pregnancy, labor, and delivery had been uneventful. He was the second of four siblings from nonconsanguineous parents. The first sibling died at age 8 months after acute episodes of metabolic acidosis, hyperammonemia, and seizures; however, screening had not revealed a specific inherited metabolic disorder.

Metabolic investigations in this patient’s first days of life revealed altered propionylcarnitine (14.2 μmol/L, normal range 0–2.99 μmol/L) and mild 3-hydroxypropionic acid increase (no value available), with associated hyperglycinemia (1645 μmol/L, normal range 60–404 μmol/L) and hyperammonemia (371 μmol/L), leading to the diagnosis of PA. Molecular analysis revealed compound heterozygosity at the propionyl-CoA carboxylase beta unit (*PCCB*) gene locus, with two pathogenic alleles: (1) G188R (c.562G>A; NCBI reference sequence NM_001178014.1), associated with the absence of detectable protein, and (2) G112D (c.335G>A, NCBI reference sequence NM_001178014.1), which produces an immunoreactive β-subunit protein of unknown functionality [6].

Oral feeding was stopped, and intravenous therapy for hyperammonemia and metabolic acidosis was initiated. The infant began a protein-restricted diet, supplemented with carnitine 100 mg/kg, biotin 5 mg/day, and metronidazole 10 mg/kg. His protein intake, between episodes of decompensation, was approximately 0.8 g/kg/day (0.2 g/kg/day as propiogenic amino acid-free formulas) from 1 year of age (protein intake by age is reported in Table 1). An adequate energy intake was maintained, avoiding prolonged fasting and supplementing the diet with single amino acids (isoleucine and valine), based on the Food and Agriculture Organization of the United Nations/World Health Organization/United Nations University (2017) guidelines [4].

The clinical course of PA was characterized by frequent severe episodes of decompensation, often triggered by infections and/or food refusal, characterized by elevated ammonia levels (> 120 μmol/L, normal range 50–80 μmol/L), severe metabolic acidosis, and frequent vomiting with or without impaired consciousness. Psychological assessments with the Portage method and Learning Accomplishment Profile highlighted cognitive impairment and delayed language development (functional development 30–36 months vs. chronological age 7 years). The patient’s stature-for-age and weight-for-age were below the normal range (Table 2), and his bone density was in the osteoporotic range (mean bone mineral density 0.63 g/cm^2^, Z-score − 2.7). No cardiac abnormalities or hematologic alterations were present, and the results of ophthalmologic tests were normal. Brain magnetic resonance imaging did not detect any morphologic abnormalities, and spectroscopy highlighted neuronal injury as an abnormal *N*-acetylaspartate/creatine ratio.

Persistent hyperammonemia and frequent metabolic attacks prompted recurrent administration of ammonia scavengers (sodium phenylbutyrate, sodium benzoate, and arginine hydrochloride). The patient was admitted 78 times for acute attacks during the first 9 years of his life, with 7–10 admissions per year (Table 1).

In view of the frequency of metabolic events with abnormal ammonia levels, we introduced at 9 years of age continuous oral administration of NCG at a dose of 100 mg/kg/day, although 6 months later we reduced the daily NCG dose to 50 mg/kg/day. Since initiation of NCG therapy, the patient’s mean ± standard deviation (SD) plasma ammonia levels have decreased significantly (75.7 ± 37 μmol/L vs. 140.3 ± 47.2 μmol/L before NCG therapy, *p* < 0.005; 18 vs. 11 measurements for before vs. after initiation of NCG therapy) (Table 2). His plasma levels of alanine remained normal throughout the treatment period, whereas glutamine levels were low before and during NCG treatment (296–297 μmol/L vs. normal range 420–730 μmol/L) (Table 2). His glycine levels were high compared with normal levels prior to NCG treatment and increased further during NCG treatment (561.8 vs. 1349 μmol/L, normal range 60–404 μmol/L) (Table 2).

Over the last 6 years, the patient experienced only two episodes of acute decompensation requiring hospitalization, both of which occurred during the first year after initiating NCG therapy; in comparison, he had eight or nine annual admissions for acute attacks in the 3 years immediately prior to the initiation of NCG therapy (Table 1). Ammonia scavengers were gradually stopped, whereas carnitine and metronidazole doses were adapted on the basis of acylcarnitine and organic acid levels. Total protein intake increased gradually, and it was 1.2 g/kg/day at the patient’s last assessment. No cardiomyopathy was detected over time, and bone density values improved from osteoporotic to osteopenic (mean bone mineral density 0.8 g/cm^2^; Z-score − 1.3).

An improvement in feeding, with less fastidious eating habits and no further cognitive deterioration (intelligence quotient 35) were detected at the most recent neurocognitive assessment (Wechsler Intelligence Scale for Children, Fourth Edition). Adherence to therapy and adverse drug reactions were regularly assessed during biochemical and clinical evaluations. Neither clinically significant adverse events nor adherence issues were reported over time.

**Table 1:** Number of admissions, mean ammonia levels, and protein intake, according to patient age

Age (years)	Number of admissions	Ammonia levels^a^, mean (±SD) (μmol/L)	Protein (natural proteins) intake (g/kg/day)
0–1	9	121 ± 14.7	1.5 (1)
1–2	10	116 ± 35.4	1.6 (0.9)
2–3	8	131 ± 12.4	1.4 (0.8)
3–4	10	89 ± 17.9	1.1 (0.7)
4–5	7	128 ± 27.3	1 (0.6)
5–6	8	96 ± 17.7	1 (0.5)
6–7	9	112 ± 30.5	1.1 (0.6)
7–8	9	199 ± 70.6	0.95 (0.6)
8–9	8	114 ± 31.8	0.9 (0.5)
9^b^–10	2	69 ± 21.5	1 (0.7)
10–11	0	74 ± 30.4	1.1 (0.8)
11–15	0	61.7 ± 48	1.2 (1)

*Abbreviations*: *NCG* N-carbamylglutamate, *SD* standard deviation

^a^Normal range 50–80 μmol/L

^b^Continuous treatment with oral NCG 100 mg/kg/day (50 mg/kg/day after 6 months) initiated

**Table 2:** Protein and energy intake, body mass index, and plasma metabolites before and after initiating continuous treatment with *N*-carbamylglutamate

Parameter	Before NCG (mean ± SD)	During NCG (mean ± SD)	*p* Value (two-tailed paired *t* test)
Natural protein intake (g/kg/day)	0.7 ± 0.12	0.85 ± 0.18	n.s.
Energy intake (kcal/kg)	67.3 ± 13.2	56.2 ± 19.1	n.s.
BMI (kg/m^2^)	14.3 (< 10th percentile)	18.4 (> 25th percentile)	n.s.
Metabolites (μmol/L, number of measurements)			
Ammonia^a^	140.3 ± 47.2, 18	75.7 ± 37, 11	0.003
Glutamine^b^	297.3 ± 64.8, 8	296 ± 160, 12	0.4
Alanine^c^	430.3 ± 123.9, 5	596.8 ± 318.9, 10	0.09
Glycine^d^	561.8 ± 496.2, 7	1349 ± 496.9, 12	0.01

*Abbreviations: BMI* Body mass index, *NCG N*-carbamylglutamate, *n.s.* Not significant, *SD* standard deviation

^a^Normal range 50–80 μmol/L

^b^Normal range 420–730 μmol/L

^c^Normal range 142–421 μmol/L

^d^Normal range 60–404 μmol/L

## Discussion

Currently, long-term treatment of PA consists of maintaining a protein-restricted diet supplemented with propionic acid precursor-free amino acids, vitamins, and minerals; administering antibiotics and carnitine; and avoiding catabolism [4]. Ammonia scavengers are not recommended for long-term therapy because of the risk of accumulating CoA esters and depleting free CoA. The use of sodium phenylbutyrate is even more controversial, owing to the risk of depleting the glutamine/glutamate pool [4]. Nevertheless, this class of medications still represents the main option for the long-term treatment of hyperammonemia secondary to organic acidemias.

There are reports on the rapid amelioration of patients’ clinical condition with precocious administration of NCG, regardless of the extent of hyperammonemia [7–11], suggesting that continuous NCG treatment may be effective. We report our highly positive clinical experience with continuous administration of NCG for 6 years in a patient with PA who presented frequently with hyperammonemia believed to reflect secondary blockage of NAG synthase by PA metabolites [2, 3]. Treatment was associated with a decrease in plasma ammonia levels and a durable reduction in the frequency of metabolic decompensations. There were no clinically significant adverse reactions to therapy or evidence of progression of multiorgan involvement. Glutamate levels were consistently low, reflecting a deficiency in tricarboxylic acid intermediates [12, 13], and glycine levels were characteristically high, likely due to effects of PA on amino acid metabolism such as the glycine cleavage system [14–16]. The observed increase in mean plasma glycine levels (Table 2) may have resulted from discontinuation of the ammonia scavenger sodium benzoate several months after starting NCG treatment.

This report on the favorable use of NCG in a patient with PA has the limitations of a single experience in terms of reliability and generalizability. Furthermore, the reduced incidence of concomitant illness and improved feeding typically observed in late childhood may have contributed to the patient’s improvement.

However, this case report describes a longer follow-up of NCG treatment (6 years) than previously in published reports (i.e., 7–16 months and 15 days for Burlina *et al*. [9] and Valayannopoulos *et al*. [10], respectively) [9, 10] and derives from observation over the pubertal period, when hormonal changes and higher growth rate make the metabolic equilibrium more unstable than in other age groups.

## Conclusions

Our observations of our patient suggest that patients with hyperammonemic decompensations secondary to PA may benefit from continuous administration of NCG. If this observation is confirmed, well-designed clinical trials should address dosage requirements and effects on concomitant therapies and protein tolerance.

## Abbreviations

BMI: Body mass index
CoA: Coenzyme A
NAG: *N*-acetylglutamate
NCG: *N*-carbamylglutamate
PA: Propionic acidemia
